# 

**Published:** 1903-07

**Authors:** 


### LITERARY NOTES.

The Batavia Woman's Hospital Association, the corporate body under the auspices of which the Batavia hospital is managed, has issued its first report which embraces the period from May, 1900, to July 15, 1902. The report shows in detail the splendid work that has been done in establishing one of the finest small hospitals in Western New York, which is a credit to every person who has contributed time, material, or money toward its completion and maintenance.

Battle & Company, of Saint Louis, have issued chart No. 2, illustrating fractures of long bones. This is the last of the series and presents Colles's fracture—fracture of the lower end of the radius—in a graphic manner. The contraction of the supinator longus acts to tilt the fracture against and thus displace the lower end of the ulna. This could have been made a more prominent feature of the drawing; nevertheless, it is an instructive delineation of this classic injury.

